# Pharmacological Cardioversion with Cavutilide: Results of Restoration and Maintenance of Sinus Rhythm in Certain Categories of Patients with Atrial Fibrillation and Atrial Flutter

**DOI:** 10.3390/jcm15072719

**Published:** 2026-04-03

**Authors:** Ekaterina Ragozina, Kristina Adushkina, Olga Sinkevich, Diana Gabidullova, Galina Fedorova, Stepan Polyakov, Dmitry Duplyakov

**Affiliations:** 1 Samara Regional Cardiology Dispensary Named After V. P. Polyakov, Samara 443070, Russia; ragozina219@gmail.com (E.R.); mkn230590@mail.ru (K.A.); sow1982@inbox.ru (O.S.); dianagabidullova@mail.ru (D.G.); galina16300@mail.ru (G.F.); 2 Department of Internal Medicine & Cardiology, Samara State Medical University, Samara 443099, Russia

**Keywords:** Cavutilide, atrial fibrillation, atrial flutter, pharmacological cardioversion

## Abstract

**Background/Objectives**: Atrial fibrillation (AF) and atrial flutter (AFl) are common arrhythmias associated with significant morbidity and mortality. Pharmacological cardioversion (PCV) is an alternative to electrical cardioversion (ECV), particularly in patients with contraindications or prior unsuccessful ECV. This study aimed to evaluate the short- and long-term efficacy and safety of Cavutilide for PCV in patients with AF/AFl, including those with obesity and previous failed ECV. **Methods**: In this prospective, open-label, single-center study, 193 consecutive patients with paroxysmal or persistent AF/AFl underwent PCV with intravenous Cavutilide (maximum total dose 30 µg/kg). Patients were stratified into three groups: obesity (*n* = 56), prior unsuccessful ECV (*n* = 50), and control (*n* = 87). Sinus rhythm (SR) restoration, early recurrence (within 24 h), adverse events, and long-term recurrence rates were assessed during a follow-up period of up to 19 months. **Results**: SR was restored in 89.6% of patients overall, including 87.5% in obese patients and 78% in those with prior failed ECV. The first dose was effective in 49.2% of cases. Early recurrence occurred in 5.2% of patients. During follow-up, AF/AFl recurrence was documented in 33.7% of patients, most frequently within the first 3 months. Restoration of SR with the lowest dose (10 µg/kg) was associated with a higher recurrence risk (OR 1.92; 95% CI 1.01–3.71; *p* = 0.03). Serious adverse events were infrequent; torsades de pointes occurred in 1.5% of patients. **Conclusions**: Cavutilide is effective for pharmacological cardioversion of AF/AFl, including in patients with obesity and prior unsuccessful ECV, with an acceptable safety profile. Careful monitoring is warranted due to the risk of proarrhythmic events and arrhythmia recurrence, particularly in the early post-cardioversion period.

## 1. Introduction

Atrial fibrillation (AF) and atrial flutter (AFl) are the most clinically significant forms of cardiac arrhythmia. The prevalence of AF in the adult population is estimated at 2–4%, reaching 10% in older age groups [1,2]. It is believed that 11% of patients with AF are asymptomatic or minimally symptomatic, remaining undetected for a long time [3]. The prevalence of AFl in the general population is significantly lower, at 0.4–0.7% [4].

AF is the reason for hospitalization of approximately one-third of patients with cardiac arrhythmias [5]. As the population ages, most countries experience an increase in the incidence of AF, and the number of hospitalizations for patients with AF/AFl increases accordingly [6,7]. In the Russian Federation, more than 70% of patients with AF repeatedly seek emergency medical care within a year [8]. The risk of cardioembolic stroke and cardiovascular death in AF increases three–five-fold [1,9].

The management of patients with paroxysmal and persistent forms of AF/AFl is a complex task, requiring the selection of a strategy aimed at alleviating the severity of symptoms and preventing severe complications. Electrical (ECV) and pharmacological cardioversion (PCV) are recognized strategies for restoring sinus rhythm (SR). The advantages and limitations of each method have been adequately studied, and the choice between them is based on the clinical presentation of the disease and existing contraindications [1].

To reduce the incidence of complications and further optimize the treatment of AF/AFl, new approaches are being developed, and the development of new antiarrhythmic drugs (AADs) continues. In 2020, a new Class III AAD, Cavutilide, was included in the National Clinical Guidelines for the Treatment of Atrial Fibrillation and Flutter [10]. Studies have demonstrated its high efficacy [11,12,13]. Therefore, analyzing the effectiveness of restoring and maintaining sinus rhythm during CV with Cavutilide in certain patient categories is relevant.

The aim of the study was to evaluate short-term and long-term outcomes of pharmacological cardioversion using Cavutilide in two groups of patients: those with obesity and those after ineffective electrical cardioversion.

## 2. Materials and Methods

This open-label prospective study included 193 consecutive patients hospitalized from March 2022 to June 2023 at the Samara Regional Clinical Cardiology Dispensary named after V.P. Polyakov with a confirmed diagnosis of paroxysmal or persistent AF and AFl. All of them underwent cardioversion with Cavutilide.

If the arrhythmia episode lasted less than 48 h, cardioversion was performed urgently. If AF lasted more than 48 h, transesophageal echocardiography (TEE) was performed to exclude thromboembolic complications. Exclusion criteria included contraindications to the use of Cavutilide: heart failure (III-IV NYHA); acute coronary syndrome; bradystolic AF and AFl with a heart rate <50 bpm or pauses >3 s; hypokalemia, hypomagnesemia, and others.

The recommended schedule of PCV was three sequential intravenous administrations of 10 µg/kg each with 15 min intervals, under continuous monitoring and ECG recording to assess the QT interval after each administration. The injection was stopped in the event of: restoration of heart rate; a decrease in heart rate to <50 bpm; an increase in the QT interval to >500 ms; and the development of proarrhythmic effects. The maximum total dose was 30 µg/kg. After PVC, patients were monitored for 24 h. Three groups were identified from a total cohort of 193 patients:

Group 1—56 patients with stage II and III obesity (body mass index ≥35 kg/m^2^), including 25 (44.6%) stage II patients and 31 (55.4%) stage III patients;

Group 2—50 patients who initially underwent ECV but failed to restore SR. Patients with unsuccessful ECV and obesity (16 patients) were assigned to Group 1, as obesity is a determining factor in the reduction in ECV effectiveness.

Group 3—a control group—87 patients without obesity and/or unsuccessful ECV.

The recurrence rate of AF/AFl was assessed for 19 months after PCV. During this period, 65 patients were readmitted, according to the established Samara City District patient routing for cardiac arrhythmias.

Statistical analysis was performed using Python 3.9. Normality was tested using the Kolmogorov–Smirnov test. Normally distributed data are presented as M ± SD; nonparametrically distributed data are presented as Me [Q25; Q75]. For pairwise group comparisons of normally distributed quantitative variables, Student’s *t*-test was used under conditions of equal variances; the Welch test was used under conditions of unequal variances. If the hypothesis of normal distribution was not met, the Mann–Whitney test was used. Pairwise group comparisons for qualitative variables were performed using the chi-square test. Differences were considered statistically significant at *p* ≤ 0.05.

To plot the recurrence rate histogram, kernel density estimation was used—a data smoothing technique that allows one to obtain an estimate of the true distribution density as a graph of a function based on empirical data. The dynamics of SR recovery after Cavutilide administration in different groups were analyzed using Barnard’s exact test.

Fisher’s exact test was used to assess the relationship between the number of doses and the occurrence of recurrence, and the odds ratio test was used to determine a direct or inverse relationship. The relationship between the number of doses and SR recovery was assessed using a logistic regression model.

## 3. Results

The average age of patients included in the study was 63.9 ± 10.9 years, and 53% were women. AF was diagnosed in 147 patients (76.2%), atrial flutter in 46 (23.8%), and paroxysmal AF/AFl was diagnosed in more than one-third of patients (35.2%). The clinical characteristics of the patients are presented in Table 1.

A characteristic feature of patients with AF is the presence of concomitant cardiovascular pathology. The most common causes of AF were hypertension, coronary artery disease (CAD), and chronic heart failure (CHF). Twenty-five patients (12.9%) had previously had a myocardial infarction, and 11.4% had undergone radiofrequency catheter ablation. Comparative characteristics of all three groups are presented in Table 2.

Patients in all three groups did not differ significantly by gender and age. Most patients had preserved LV systolic function and mild LA dilation. A more pronounced degree of LA dilation was observed in Group 1, where more than 55% of patients had class III obesity, weighing over 100 kg. In addition, every fourth patient had diabetes. This group, as noted above, included patients who failed to restore heart rhythm with ECV (16 patients, including 5 patients who failed to normalize their rhythm with either ECV or PCV).

Overall, after PCV with Cavutilide, heart rhythm was restored in 87.1% of patients with AF (92.3% with paroxysmal AF and 85.2% with persistent AF) and in 97.8% with AFl (96.2% with paroxysmal AFl and 100% with persistent AFl).

**Figure 1:**
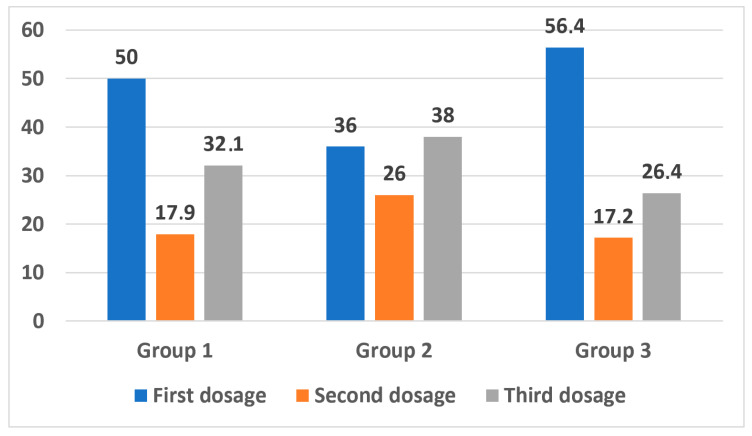
Sinus rhythm restoration depending on the drug dose.

The first dose of the drug restored SR in almost half of the patients—49.2%; the second bolus restored SR in an additional 19.7%. The maximum total dose of 30 µg/kg restored SR in 31.1% of patients. The results of SR restoration depending on the drug dose by group are presented in Figure 1.

In the comparison group, SR was restored after the first bolus in 56.4% of patients, and only 26.4% required all three doses of the drug. The minimum dose of Cavutilide (10 µg/kg) was the least effective in restoring SR in patients who had previously undergone ECV—36% versus 56.4% in the comparison group (*p* = 0.027). The second bolus of the drug in the ECV group was more effective—SR was restored in 26% of patients versus 17.9% and 17.2% in groups 1 and 3; however, the differences were not statistically significant. In the ECV group, SR was restored after a total dose of 30 µg/kg in 38% of patients, compared to 32.1% and 26.4% in groups 1 and 3, respectively.

Twenty patients (10.4%) failed to restore SR; more than half (11) of these cases were in group 2, despite initial ECV and subsequent PCV.

After SR restoration with PCV, early (within 24 h) AF recurrences occurred in 10 patients (5.2%). These included four patients after the minimum dose (10 µg/kg), five after two doses (20 µg/kg), and only one patient after three doses (30 µg/kg). The use of the maximum dose of Cavutilide is less likely associated with the development of early AF/AFl recurrences.

During PCV, asymptomatic sinus bradycardia developed in 21 patients (9%) within 24 h after drug administration, with pauses longer than 3.0 s recorded in two cases (1%). Non-sustained episodes of monomorphic ventricular tachycardia that did not require medication occurred in 6 patients (3.1%). Asymptomatic prolongation of the QT interval by more than 500 ms after drug administration was recorded in 32 patients (17%).

In three cases (1.5%), a serious complication, torsades de pointes (TdP), developed alongside a prolonged QT interval, requiring emergency ECV. Two cases were recorded after a single bolus and one after three doses of the drug. Patient #1: M63 years, no structural heart disease (EF 54%), QTc before PCV 378 ms, QT after PCV—505 ms, time to TdP—9 min after first intravenous administrations of 10 µg/kg. Patient #2: F65 years, no structural heart disease (EF 64%), QTc before PCV 404 ms, QT after—524 ms, time to TdP 12 min after first intravenous administrations of 10 µg/kg. Patient #3: M64 years, no structural heart disease (EF 58%), QTc before PCV 386 ms, QT after PCV—556 ms, time to TdP—9 min after third intravenous administrations of 30 µg/kg. All of them had a persistent type of AF with a duration over 1 year and fairly normal kidney function (GFR over 60 mL/min/1.73m^2^). Two of these patients experienced recurrences of TdP 10 h after the first dose (patient#2) and 12.5 h after three doses of the drug (patient #3), requiring repeated ECV.

Upon discharge, all patients were prescribed antiarrhythmic therapy in accordance with current clinical guidelines. More than one-third of patients were receiving sotalol, more than 18% were taking metoprolol, and 20% were receiving propafenone. Bisoprolol was recommended as monotherapy and in combination with amiodarone in 25% of patients.

**Figure 2:**
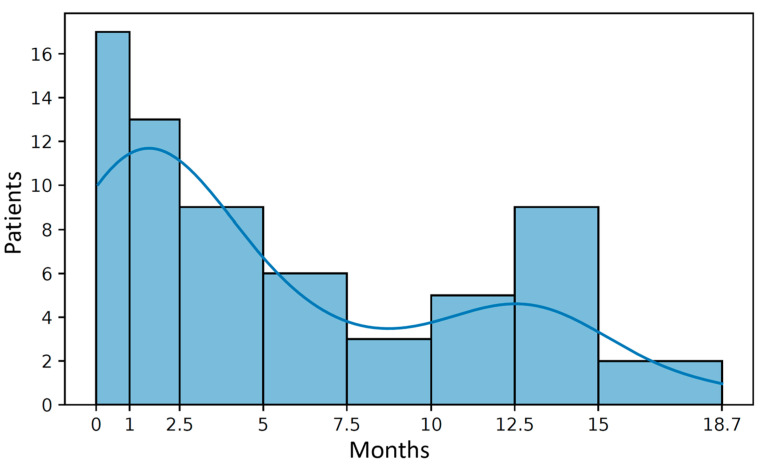
AF/AFl recurrence rate after Cavutilide cardioversion.

During 19 months of follow-up, 65 (33.7%) cases of documented AF/AFl recurrences occurred, including 22 patients who were hospitalized twice, and four patients who experienced three episodes each. Recurrences were more common in women—61.5%. The AF/AFL recurrence rate in patients in Group 3 was 42.5%, while in the other two groups it was 28.6% and 24%, respectively (*p* = 0.046). More than half (52.3%) of AF/AFl recurrences occurred in the first three months after PCV, and 78.5% of all recurrences identified during the follow-up period occurred within a year (Figure 2).

**Figure 3:**
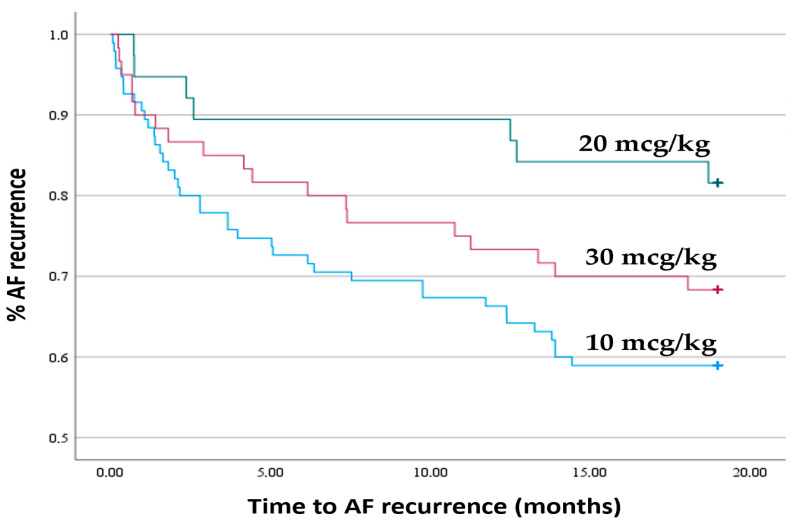
Kaplan–Meier recurrence curve depending on the dose of Cavutilide (log-rank test).

Statistical analysis of the relationships between the incidence of recurrence and the dose of Cavutilide revealed the strongest association between the occurrence of arrhythmia recurrence and the administration of the lowest dose of the drug, after which cardiac rhythm was restored. With the lowest dose, the likelihood of recurrence was almost twice as high as in the other two cases (Table 3). The Kaplan–Meier recurrence curve depending on the dose of Cavutilide is presented in Figure 3 (Log rank-test, χ^2^ = 6.368, df = 2, *p* = 0.041). Patients who restored rhythm after the minimum dose (10 mcg/kg) had a statistically significantly higher risk of recurrence compared to patients who required the medium dose (20 mcg/kg) (*p* = 0.015). Differences between other pairs of groups did not reach statistical significance.

## 4. Discussion

According to studies, the efficacy of Cavutilide cardioversion is comparable to that of ECV [11,12]. However, when concluding that the two methods are equivalent, it is important to consider the broad range of contraindications associated with antiarrhythmic drugs but absent with ECV.

The patient groups identified in our study—those with obesity and those after unsuccessful ECV—reflect the actual patient population with AF/AFl that has developed in clinical practice. The results of the study demonstrated a high efficacy of 89.6% for drug-induced cardioversion using Cavutilide in paroxysmal and persistent atrial fibrillation and atrial flutter, as well as a relatively high safety profile—the incidence of severe complications such as TdP was 1.5%. However, the recorded recurrences of TdP at 6 and 12.5 h after PCV suggest the need for 24 h observation of patients in the intensive care unit.

In the comparison group without obesity and ineffective ECV, Cavutilide successfully restored SR in 56.4% of cases after the first dose; only 2 patients (2.3%) failed to restore SR after PCV.

In the obese group, the effectiveness of PCV was 87.5%. These patients were characterized by pronounced left heart remodeling. It is well known that ECV terminates AF in only 25% of cases with body weight exceeding 100 kg [14]. In our study, SR was restored in 90.3% (28 of 31) of patients with AF of similar weight. The success rate of Cavutilide PCV in 16 patients in this group with a history of initially unsuccessful ECV was 68.8%.

Currently, data have been published on the successful implementation of Cavutilide PCV after unsuccessful ECV attempts in 25 patients with stage II and III obesity [13,15]. The results of our study confirm that Cavutilide can be considered as a first-line agent for restoring sinus rhythm in this group [16].

Patients with unsuccessful ECV comprised 34.2% of all patients included in the study. The presence of such a large category of patients reflects real-world clinical practice and should guide physicians choosing the cardioversion method to the need for a detailed analysis of the patient’s medical history. Patients included in this group were characterized by a reduced LV EF (*p* = 0.03) relative to the comparison group and a lower probability of restoring SR with a single minimal dose of Cavutilide (*p* = 0.027). It is known that as the duration of continuous AF increases, the effectiveness of ECV decreases. The duration of arrhythmia in patients with unsuccessful ECV in our study was 4.7 ± 5.1 years and was longer than in other groups, although the differences did not reach the level of statistical significance.

The largest proportion of patients (22% of ECV patients, 11 of 50) failed to restore sinus rhythm, significantly higher than in the comparison group (*p* = 0.0005). The effectiveness of PCV was 78%. Taking into account obese patients with ineffective ECV included in Group 1, this result drops to 75.8%. Dzaurova H.M. et al. demonstrated similar effectiveness of PCV with Cavutilide in such patients—85.4% [13].

It should be noted that of the 20 patients who failed to restore sinus rhythm after PCV with Cavutilide, 80% (16 patients) initially underwent failed ECV. It has been shown that cases of ineffective use of both types of cardioversion (ECV and PCV) to terminate AF/AFl may be genetically determined [17]. This may be considered an indication to change the management strategy for these patients to heart rate control.

Our study presents the first long-term results of PCV with Cavutilide. During 19 months of follow-up after PCV, recurrence of AF/AFl was documented in 65 patients (33.7%), more than one-third of whom were hospitalized twice. Recurrences of AF/AFl were most frequently recorded within 3 months after PCV (52.3%), and in the first 30 days (26.1%). A similar trend is characteristic of recurrences after ECV [18]. 78.5% of all identified cases of recurrence were recorded within 1 year. According to the literature, the recurrence rate of AF within 1 year after ECV can also be up to 80% [19]. Recurrences occur more frequently in women—61.5% of cases, which confirms existing data [18,20].

The vast majority of our patients had mild LA dilation with its higher degree observed in Group 1, where more than 55% of patients had class III obesity, weighing over 100 kg. However, not only LA size, but such functional parameters like left atrial reservoir strain may have prognostic value. Recent studies have demonstrated that reduced left atrial reservoir strain is associated with a higher risk of AF recurrence after electrical cardioversion and catheter ablation [21,22].

For the first time, an interesting relationship was identified between arrhythmia recurrence and the drug dose received, after which the rhythm was restored. After arrhythmia was terminated with a single dose of the drug, a 60% recurrence rate was observed. With a Cavutilide dose of 10 µg/kg, sufficient to restore rhythm, the chance of recurrence was almost 2 times higher (OR 1.92; 95% CI 1.0075–3.71; *p* = 0.03) than after administration of a total dose of 30 µg/kg (OR 0.88; 95% CI 0.43–1.75; *p* = 0.74). Moreover, the highest recurrence rate in the control group was noted despite better intracardiac hemodynamic and cardiac geometry parameters, which differ significantly from echocardiography parameters in obese patients.

TdP occurred in 1.5% of patients with AF on Cavutilide in our study, and progression from QTc prolongation to TdP development was observed in 9% of them. Wongsalap Y et al. was observed in 33 (1.12%) cases of intravenous amiodarone-associated QTc interval prolongation (among 2944 patients with AF), and TdP occurred in 16 (50%) of them [23]. Of 2036 patients with AF treated with Dofetilid, 105 patients experienced dofetilide-related QTc prolongation (incidence rate 5.2%), and 16 of them suffered TdP (15.3%) [24]. A meta-analysis of safety data of Ibutilide showed an overall incidence of TdP of 4.3% in patients with AF, with a 1.7% chance of a sustained TdP requiring ECV, but no reported deaths or severe morbidity [25].

Two patients had recurrent TdP episodes 10–12.5 h after Cavutilide was given. One patient had it 10 h after the first dose, while for another, it was 12.5 h after three full doses of the drug. Presumably, such proarrhythmic effects may have some genetic background [26,27] and should be considered as drug-induced [28]. A longer observation period is reasonable when Cavutilide is used for PCV.

## 5. Limitations

This study has several limitations: (1) the presented data are obtained from a single-center study, which limits its external validity; (2) it is a non-randomized study that might have selection bias and confounding; and (3) there was no direct comparator arm with ECV alone or any other medicines.

## 6. Conclusions

Cavutilide is highly effective and safe in restoring sinus rhythm in patients with paroxysmal and persistent AF/AFl with grade II and III obesity and a history of previous unsuccessful ECV.

## Figures and Tables

**Table 1:** Clinical characteristics of the patients.

Characteristics	Value (*n* = 193)
Age, years	63.9 ± 10.9
Gender male/female, *n* (%)	91 (47.2)/102 (52.8)
Atrial fibrillation, *n* (%)	147 (76.2)
Atrial flutter, *n* (%)	46 (23.8)
Paroxysmal AF/AFl, *n* (%)	68 (35.2)
Persistent AF/AFl, *n* (%)	125 (64.8)
Duration of persistent AF/AFl episode, days	107.8 ± 87.1
Duration of paroxysmal AF/AFl episode, h	34.1 ± 26.4
Duration of AF/AFl history, months [25; 75]	30.6 [10; 68.8]
History of RFA, *n* (%)	22 (11.4)
CHF I-II NYHA, *n* (%)	32 (16.6)
Hypertension, *n* (%)	172 (89.1)
CAD, *n* (%)	42 (21.8)
Previous MI, *n* (%)	25 (12.9)
COPD, *n* (%)	11 (5.7)
DM, *n* (%)	34 (17.6)

Note: RFA—Radiofrequency Catheter Ablation; CHF—Chronic Heart Failure, CAD—Coronary Artery Disease, MI—Myocardial Infarction, COPD—Chronic Obstructive Pulmonary Disease, and DM—Diabetes Mellitus.

**Table 2:** Clinical and demographic characteristics of patients by group.

Indicator	Group 1 Obesity (*n* = 56)	Group 2 Ineffective ECV (*n* = 50)	Group 3 Comparison (*n* = 87)	Pairwise *p*-Test (1:2, 1:3, 2:3)
Age (years), M ± SD	62.9 ± 9.3	63.1 ± 11.0	65.2 ± 11.8	0.94 * 0.23 * 0.30 *
Women, *n* (%)	33 (58.9)	23 (46.0)	46 (52.4)	0.25 ^‡^ 0.59 ^‡^ 0.55 ^‡^
Atrial flutter, *n* (%)	15 (26.8)	5 (10.0)	26 (29.9)	0.0504 ^‡^ 0.83 ^‡^ 0.01^‡^
AF/AFl Duration (months), Me [Q25; Q75]	25 (6; 60.8)	36.5 (14.1; 85.2)	30.4 (9.9; 60.8)	0.22 ^†^ 0.79 ^†^ 0.33 ^†^
Hypertension, *n* (%)	54 (96.4)	47 (94.0)	71 (81.6)	0.90 ^‡^ 0.02 ^‡^ 0.08 ^‡^
CHF I-II NYHA, *n* (%)	11 (19.6)	9 (18.0)	12 (13.8)	1.0 ^‡^ 0.48 ^‡^ 0.17 ^‡^
CAD, *n* (%)	7 (12.5)	8 (16.0)	27 (31.0)	0.81 ^‡^ 0.02 ^‡^ 0.08 ^‡^
Previous MI, *n* (%)	5 (8.9)	8 (16.0)	12 (13.8)	0.42 ^‡^ 0.54 ^‡^ 0.92 ^‡^
DM, *n* (%)	14 (25.0)	9 (18.0)	11 (12.6)	0.52 ^‡^ 0.09 ^‡^ 0.55 ^‡^
LA diameter (cm), Me [Q25; Q75]	4.4 [4; 4.8]	4.1 [3.8; 4.5]	4.2 [3.8; 4.5]	0.007 ^†^ 0.004 ^†^ 0.81 ^†^
LA volume (ml), Me [Q25; Q75]	56 [50; 7]	60 [52.3; 67.8]	56 [45; 69]	0.54 ^†^ 0.49 ^†^ 0.30 ^†^
LVEDD (cm), Me [Q25; Q75]	5.2 [4.8; 5.6]	5 [4.6; 5.5]	5 [4.5; 5.6]	0.22 ^†^ 0.04 ^†^ 0.63 ^†^
IVS (cm), Me [Q25; Q75]	1.1 [1; 1.3]	1 [0.9; 1.1]	1 [0.95; 1.2]	0.048 ^†^ 0.04 ^†^ 0.93 ^†^
LVEF, % Me [25; 75]	60 [53.5; 67.5]	57 [53.3;61.5]	61 [55; 66]	0.26 ^†^ 0.59 ^†^ 0.03 ^†^
SR not restored, *n* (%)	7 (12.5)	11 (22.0)	2 (2.3)	0.3 ^‡^ 0.06 ^‡^ 0.0005 ^‡^
Recurrence after discharge, *n* (%)	16 (28.6)	12 (24.0)	37 (42.5)	0.75 ^‡^ 0.13 ^‡^ 0.046 ^‡^

Note: Pairwise *p*-value test by groups is presented in the following sequence: 1:2; 1:3; 2:3. Legend: *—according to Student’s *t*-test; ^†^—according to the Mann–Whitney U-test; ^‡^—according to the chi-square test. CHF—chronic heart failure, CAD—coronary artery disease, MI—myocardial infarction, DM—diabetes mellitus, LA—left atrium, EDD—end-diastolic dimension, LV—left ventricle, IVS—interventricular septum, EF—ejection fraction, and SR—sinus rhythm.

**Table 3:** Rate of post-hospital AF/AFl recurrence depending on the dose of Cavutilide.

Dose	Cases, *n* (%)	*p* *	OR **	95% Confidence Interval
10 mcg/kg	39 (60)	0.03	1.92	(1.0075; 3.71)
20 mcg/kg	7 (10.8)	0.03	0.38	(0.13; 0.95)
30 mcg/kg	19 (29.2)	0.74	0.88	(0.43; 1.75)

Note: * Fisher’s exact test; ** OR—odds ratio.

## Data Availability

The original materials presented in this trial are included in the article; further inquiries can be directed to the corresponding authors.

## Abbreviations

The following abbreviations are used in this manuscript:

AADs	Antiarrhythmic drugs
AF	Atrial fibrillation
AFl	Atrial flutter
CAD	Coronary artery disease
CHF	Chronic heart failure
COPD	Chronic obstructive pulmonary disease
DM	Diabetes mellitus
ECV	Electrical cardioversion
EDD	End-diastolic dimension
EF	Ejection fraction
IVS	Interventricular septum
LV	Left ventricle
MCV	Medical cardioversion
MI	Myocardial infarction
OR	Odds ratio
RFA	Radiofrequency catheter ablation
SR	Sinus rhythm
TEE	Transesophageal echocardiography
